# Supplementary material for: An Overview of Clinical Manifestations of Dermatological Disorders in Intensive Care Units: What Should Intensivists Be Aware of?
Source: Diagnostics (Basel). 2023 Mar 29;13(7):1290. doi: 10.3390/diagnostics13071290 (PMC10093365; doi:10.3390/diagnostics13071290)
Supplement: Supplementary file 1 [file diagnostics-13-01290-s001.zip › diagnostics-2203228-supplementary.pdf]

Table S1. Classification of Dermatological Manifestations(DM) in Intensive Medicine

GROUP	
**Group 1. DMs of life-threatening skin diseases that require ICU admission.**	1.1.Pemphigus Vulgaris (PV)
	1.2.Stevens-Johnson syndrome (SJS) and Toxic epidermal necrolysis (TEN)
	1.3.Necrotizing fasciitis
	1.4.Toxic shock syndrome (TSS)
	1.5.Staphylococcal scalded skin syndrome (SSSS), or Ritter's Disease
	1.6.Meningococcemia
	1.7.Rocky Mountain spotted fever
	1.8.Exfoliative erythroderma
	1.9.Generalized pustular psoriasis (GPP)
**Group 2. DMs associated with adverse drug reactions.**	Most DISRs are presented as mild dermatitis and self-resolving as maculopapular rash, urticaria, angioedema, and erythema multiforme. Less commonly, it is life-threatening patterns that include erythroderma, Stevens–Johnson syndrome and toxic epidermal necrolysis

**Group 3. DMs associated with systemic diseases.**	**3.1. Seborrheic Dermatitis**
	**3.2. Erythema Multiforme**
	**3.3. Vitiligo**
	**3.4. Erythema Nodosum**
	**3.5. Cutaneous metastases**
	**3.6. Paget's Disease**
	**3.7. Acanthosis nigricans**
	**3.8. Sweet's Syndrome (Acute febrile neutrophilic dermatosis)**
	**3.9. Amyloidosis of the skin**
	**3.10. Erythema Gyratum Repens**
	**3.11. Cyanosis**
	**3.12. Clubbing**
	**3.13. Necrolytic Migratory Erythema (glucagonoma syndrome)**
	**3.14. Cutaneous leukocytoclastic vasculitis**
	**3.15. Lichen planus**
	**3.16. Necrolytic acral erythema**
	**3.17. Hereditary Hemorrhagic Telangiectasia (Osler-Weber-Rendu syndrome)**
	**3.18. Pyoderma Gangrenosum**
	**3.19. Nephrogenic Systemic Fibrosis**
	**3.20. Diabetes Mellitus-Related Skin Conditions**

**Group 4. DMs as a consequence of the critical illness**	**4.1. Dermatological disorders of infectious origin**
	**4.2. Dermatological disorders induced by vasopressor drugs**
	**4.3. pressure sores**
	**4.4. Traumatic lesions due to invasive ICU procedures.**
	**4.5. Contact Dermatitis**
	**4.6. Skin Necrosis associated with Disseminated Intravascular Coagulation**
